# Systemic Immune–Inflammation Index (SII) as a Predictive Biomarker of Therapeutic Response in Psoriasis: A Retrospective Comparative Analysis of Anti-TNF, Anti-IL-17, and Anti-IL-23 Agents

**DOI:** 10.3390/jpm16060323

**Published:** 2026-06-16

**Authors:** Emanuele Trovato, Francesca La Marca, Benedetta Simonini, Martina Dragotto, Enrico Calandra, Francesca Lussana, Alessandra Cartocci, Pietro Rubegni

**Affiliations:** 1 Dermatology Unit, Department of Medical, Surgical and Neurological Sciences, University of Siena, 53100 Siena, Italy; francescalamarca93@gmail.com (F.L.M.); benedettasimonini@gmail.com (B.S.); m95.dragotto@gmail.com (M.D.); enricocalandra96@gmail.com (E.C.); francescaa.lussana@gmail.com (F.L.); pietro.rubegni@unisi.it (P.R.); 2 Department of Molecular and Developmental Medicine, University of Siena, 53100 Siena, Italy; alessandr.cartocci@gmail.com

**Keywords:** psoriasis, systemic immune–inflammation index, biomarkers, inflammation, biological therapy, personalized medicine

## Abstract

**Background/Objectives:** The Systemic Immune–Inflammation Index (SII), derived from routine blood counts, has emerged as a potential marker of systemic inflammation in psoriasis. However, its longitudinal behavior across different systemic and biologic therapies remains poorly characterized. This study aimed to evaluate changes in SII over time, assess its relationship with Psoriasis Area and Severity Index (PASI) scores, and compare SII trajectories among different treatment classes. **Methods:** A retrospective single-center study included 210 adults with psoriasis treated for 12 months with cyclosporine, anti-TNF-α, anti-IL-17, or anti-IL-23 agents. SII and PASI were recorded at baseline, 16, 36, and 52 weeks. Correlations between SII and PASI were assessed using Spearman’s analysis. Longitudinal changes were evaluated using the Friedman test, and treatment-group differences were assessed using Kruskal–Wallis analysis. An adjusted multivariable linear regression model including age, sex, body mass index, psoriatic arthritis, baseline PASI, and treatment group was performed to identify factors associated with Δ%SII. **Results:** SII correlated with PASI at baseline (ρ = 0.406, *p* < 0.001) and at 52 weeks (ρ = 0.186, *p* = 0.007), whereas no significant associations were observed at intermediate timepoints. Longitudinal analyses demonstrated significant differences in SII trajectories among treatment groups (*p* < 0.001). SII increased over time in the cyclosporine and anti-TNF-α groups, while anti-IL-17 and anti-IL-23 therapies were associated with marked and sustained reductions. In the adjusted model, anti-IL-17 (β = −90.7, 95% CI −119.6 to −61.8, *p* < 0.001) and anti-IL-23 therapies (β = −97.9, 95% CI −126.2 to −69.6, *p* < 0.001) remained independently associated with greater reductions in SII compared with cyclosporine, whereas anti-TNF therapy showed no significant difference. **Conclusions:** SII is a dynamic marker of systemic inflammatory changes in psoriasis and exhibits distinct longitudinal patterns according to treatment class. The pronounced reductions observed with IL-17 and IL-23 inhibitors support the potential value of SII as an adjunctive measure of systemic inflammation. However, prospective studies are required to clarify its clinical utility and determine its role in routine patient management.

## 1. Introduction

Psoriasis is a chronic immune-mediated inflammatory disease affecting approximately 2–3% of the global population and is increasingly recognized as a systemic condition rather than a purely cutaneous disorder [1]. Over the past two decades, advances in immunology, epidemiology, and translational research have demonstrated that the inflammatory pathways active in psoriatic skin lesions are also operative at the systemic level, contributing to a broad spectrum of comorbidities and long-term health risks [2]. Beyond the skin, psoriasis is associated with multiple comorbidities, including psoriatic arthritis (PsA), metabolic syndrome, obesity, insulin resistance, non-alcoholic fatty liver disease, and cardiovascular disease [3,4,5,6,7,8]. Patients also experience increased rates of anxiety, depression, and impaired quality of life [9]. Collectively, these manifestations support the concept of psoriasis as a systemic inflammatory disorder characterized by persistent low-grade immune activation extending beyond the skin [10]. The concept of the “psoriatic march” further suggests that chronic inflammation may contribute to endothelial dysfunction, accelerated atherosclerosis, and increased cardiovascular morbidity and mortality [11,12]. In this context, the assessment of systemic inflammation remains challenging. Traditional inflammatory markers provide only a partial representation of disease activity, prompting growing interest in hematological indices derived from routine blood counts, such as the neutrophil-to-lymphocyte ratio (NLR), platelet-to-lymphocyte ratio (PLR), and the Systemic Immune–Inflammation Index (SII) [13]. Among these, SII, calculated as (neutrophils × platelets)/lymphocytes, integrates key components of innate and adaptive immunity into a single composite measure. Elevated SII values have been reported in patients with moderate-to-severe psoriasis and have been associated with disease severity, metabolic comorbidities, and overall inflammatory burden [14]. Furthermore, reductions in SII following systemic or biologic therapy suggest that this index may represent a dynamic marker of treatment-induced modulation of systemic inflammation [15]. At the immunological level, psoriasis is driven primarily by dysregulation of the IL-23/IL-17 axis, which plays a central role in sustaining keratinocyte activation and chronic inflammation [16]. This pathogenic pathway has become the main therapeutic target of modern biologic therapies and has substantially transformed the management of moderate-to-severe psoriasis. For many years, conventional systemic agents such as cyclosporine, methotrexate, and acitretin represented the cornerstone of treatment, although their long-term use is often limited by cumulative toxicity, variable efficacy, and the need for close laboratory monitoring [17,18,19,20]. The introduction of biologic therapies has revolutionized psoriasis management by selectively targeting key inflammatory mediators. Anti-tumor necrosis factor (anti-TNF) agents were the first biologics to demonstrate substantial efficacy in psoriasis and PsA, followed by therapies targeting IL-17 and IL-23, which have achieved unprecedented levels of skin clearance and durable disease control [21,22]. Despite these advances, treatment responses remain heterogeneous and a proportion of patients continue to experience suboptimal outcomes, highlighting the need for biomarkers capable of reflecting systemic inflammatory activity and therapeutic response [23]. The Psoriasis Area and Severity Index (PASI) remains the gold standard for evaluating cutaneous disease severity and treatment efficacy [24]. However, PASI primarily reflects skin involvement and may not fully capture the systemic inflammatory burden associated with psoriasis. In addition, PASI assessment requires clinical evaluation and may be subject to inter-observer variability in routine practice. Consequently, increasing attention has been directed toward simple and reproducible biomarkers that may complement clinical assessment. Originally developed in oncology, SII has emerged as a promising composite marker of systemic inflammation [25]. By combining neutrophils, lymphocytes, and platelets into a single index, SII provides a broader representation of immune activation than individual hematological parameters alone [26,27]. Preliminary studies in psoriasis have demonstrated associations between SII and disease severity, inflammatory burden, and metabolic dysfunction [28]. Nevertheless, important questions remain unanswered. It is still unclear whether SII can reliably track longitudinal changes in systemic inflammation during treatment and whether its behavior differs across therapeutic classes characterized by distinct mechanisms of action. The impact of cyclosporine, anti-TNF, anti-IL-17, and anti-IL-23 therapies on SII has not been systematically explored [29]. Because these treatments exert different immunomodulatory effects, they may influence the hematological components of SII differently and potentially generate distinct patterns of systemic inflammatory modulation [30]. Understanding these dynamics may help clarify the biological significance of SII and its potential role in clinical practice. Therefore, the aim of the present study was to evaluate longitudinal changes in SII in patients with psoriasis receiving cyclosporine, anti-TNF, anti-IL-17, or anti-IL-23 therapies in a real-world setting. We also investigated the relationship between SII and clinical disease severity, assessed through PASI, and explored whether different therapeutic classes were associated with distinct patterns of SII modulation over time. By integrating clinical and laboratory parameters, this study seeks to further define the potential role of SII as a biomarker of systemic inflammation in psoriasis.

## 2. Materials and Methods

A retrospective single-center study was conducted at the Dermatology Unit of the Hospital of Siena in accordance with the Declaration of Helsinki (1964 and subsequent amendments) and was approved by the local Ethics Committee (N° 22045). The primary objective of the study was to analyze the temporal variation of the SII in patients with psoriasis undergoing different systemic therapies. Secondary objectives included evaluating the correlation between SII and the PASI at different timepoints (baseline (T0), 16 weeks (T1), 36 weeks (T2), 52 weeks (T3)) comparing the effects of various systemic treatments (cyclosporine, anti-TNF-α, anti-IL-17, and anti-IL-23 agents) on SII trends, identifying hematological and clinical parameters predictive of a favorable inflammatory response expressed as percentage change in SII (Δ%SII). Inclusion criteria were age ≥ 18 years, confirmed diagnosis of psoriasis, continuous treatment with the same systemic drug for at least 12 months, and availability of complete laboratory data at all four predefined timepoints. Exclusion criteria included prior systemic therapy within the previous 6 months, the presence of concomitant acute infections, and active hematological or neoplastic diseases. As this was a retrospective real-world study, no formal a priori sample size calculation was performed. All consecutive patients fulfilling the inclusion criteria during the study period were included. The final sample size was therefore determined by the number of eligible patients available during the study period. At each timepoint, both clinical and laboratory data were systematically collected. Clinical evaluation included the assessment of disease severity using the PASI score and the documentation of the presence or variation of associated comorbidities. Laboratory evaluation included measurements of neutrophil, lymphocyte, and platelet counts, which were used to calculate the SII. All laboratory measurements were performed in the same hospital laboratory according to standardized institutional procedures. These parameters were longitudinally monitored to assess changes in systemic inflammatory status and treatment response, allowing for a comprehensive analysis of SII dynamics across different therapeutic classes. Additional variables collected for each patient included demographic and clinical characteristics such as age, gender, body mass index (BMI, expressed in kg/m^2^), and disease duration calculated from the time of initial diagnosis. The presence of major systemic comorbidities frequently associated with psoriasis, including PsA, arterial hypertension, type 2 diabetes mellitus, and dyslipidemia, were also recorded. Furthermore, the distribution of cutaneous involvement was documented, including nail, palmoplantar, scalp, genital, and pretibial regions, to provide a detailed and comprehensive characterization of the study population.

### Statistical Analysis

Statistical analysis was performed using IBM SPSS Statistics software (version 26.0; IBM Corp., Armonk, NY, USA). Continuous variables were expressed as mean ± standard deviation (SD) or median with interquartile range (IQR), depending on data distribution assessed by the Shapiro–Wilk test, while categorical variables were reported as absolute frequencies and percentages. As most variables did not follow a normal distribution, non-parametric tests were applied for comparisons between independent groups. Univariate analysis was conducted to evaluate relationships between key clinical and hematological parameters and included Spearman’s rank correlation coefficient (ρ) to assess the association between SII and PASI at all four timepoints (T0, T1, T2, and T3), Mann–Whitney U test to compare percentage changes in SII (Δ%SII) between two subgroups (e.g., sex, presence or absence of comorbidities, or specific disease localizations), and Kruskal–Wallis test to compare Δ%SII among the four treatment groups (A–D). When overall statistical significance was observed, post hoc pairwise comparisons were performed using Dunn–Bonferroni correction. Longitudinal analysis of SII across timepoints was performed using the Friedman test. When statistically significant, post hoc pairwise comparisons were performed using Wilcoxon signed-rank tests with Bonferroni correction to evaluate within-subject changes across timepoints (T0–T3). Between-group differences were assessed separately using the Kruskal–Wallis test. To identify independent factors associated with changes in systemic inflammation, a multivariable linear regression model was performed to identify factors associated with Δ%SII. The model included age, sex, body mass index (BMI), psoriatic arthritis, baseline PASI, and treatment group as independent variables.

## 3. Results

### 3.1. Characteristics of the Cohort

A total of 210 patients were included and stratified into four groups based on the systemic therapy received: Group A consisted of 45 patients treated with cyclosporine (21 females and 24 males; mean age 46 years); Group B included 56 patients treated with anti-TNF-α therapy (adalimumab) (27 females and 29 males; mean age 42 years); Group C comprised 54 patients treated with anti-IL-17 agents, including secukinumab (n = 28), ixekizumab (n = 13), brodalumab (n = 6), and bimekizumab (n = 7) (24 females and 30 males; mean age 55 years); and Group D included 55 patients treated with anti-IL-23 agents, including guselkumab (n = 17), risankizumab (n = 32), and tildrakizumab (n = 6) (28 females and 27 males; mean age 55 years). Overall, the study population had a mean age of 49.7 ± 17.5 years, with a balanced sex distribution (100 females and 110 males). The mean body mass index (BMI) of the cohort was 26.4 ± 4.2 kg/m^2^, indicating a tendency toward overweight; slightly higher values were observed in the anti-TNF-α group (27.7 ± 4.8), while lower mean BMI values were found in the anti-IL-17 and anti-IL-23 groups (26.4 ± 3.4 and 25.8 ± 4.2, respectively). The mean disease duration was 16.2 ± 9.5 years, consistent with a population predominantly affected by long-standing chronic psoriasis, with only minor differences across treatment groups (ranging from 15.4 ± 10.7 years in Group A to 17.2 ± 8.7 years in Group D). Regarding comorbidities, arterial hypertension was the most prevalent condition (26.2%), followed by dyslipidemia (18.1%), psoriatic arthritis (14.3%), and type 2 diabetes mellitus (13.3%). Psoriatic arthritis was absent in the cyclosporine group, whereas it was more frequently observed in patients treated with anti-TNF-α (23.2%) and anti-IL-17 agents (20.4%), consistent with their established role in joint involvement. Hypertension was relatively evenly distributed across groups (21–31%), while dyslipidemia was more common in the anti-IL-23 group (23.6%), possibly reflecting the higher mean age. Concerning disease localization, the most affected sites in the overall population were the scalp (52.4%) and palmoplantar regions (39.5%), followed by nail involvement (37.6%), genital involvement (23.3%), and pretibial localization (6.7%). Among subgroups, scalp involvement was more frequent in patients treated with anti-IL-23 (65.6%), while nail psoriasis was more prevalent in Groups A and B (44.4% and 32.1%, respectively). Palmoplantar involvement was consistently observed across all groups (33–46%), whereas genital involvement was more common in patients receiving anti-TNF-α (33.3%) and anti-IL-17 therapies (26.9%). Baseline demographic and clinical characteristics differed across treatment groups, particularly with respect to age and prevalence of psoriatic arthritis, reflecting treatment allocation in routine clinical practice. These differences should be considered when interpreting between-group comparisons. All the characteristics are reported in Table 1.

### 3.2. SII-PASI Correlations

**Figure 1:**
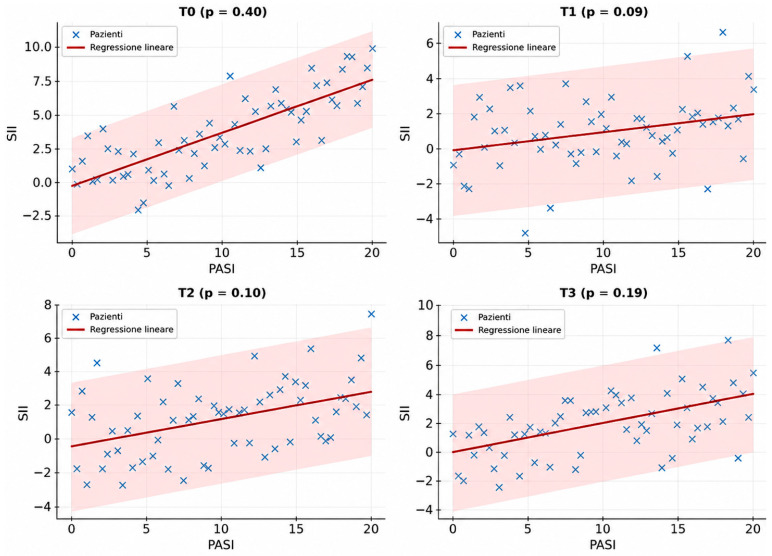
Correlation between SII and PASI at the four time points (T0–T3).

Spearman’s correlation analysis revealed a statistically significant association between SII and PASI score at two timepoints. At T0, a moderate positive correlation was observed (ρ = 0.406, *p* < 0.001), indicating that higher SII values were associated with greater disease severity as measured by PASI. At the intermediate timepoints (T1 and T2), the correlation was not statistically significant (ρ = 0.092, *p* = 0.183 and ρ = 0.108, *p* = 0.119, respectively). At T3, a weaker but still significant positive correlation was again detected (ρ = 0.186, *p* = 0.007) (Table 2, Figure 1). These findings suggest that the relationship between systemic inflammation and clinical severity is more evident at the beginning and at the end of the observation period, while it becomes less pronounced during the intermediate phases of treatment. An interesting finding was the divergent behavior observed in the cyclosporine group, where PASI markedly improved despite a progressive increase in SII. This observation suggests that SII does not necessarily parallel cutaneous response under all therapeutic conditions. Because SII is derived from neutrophil, platelet, and lymphocyte counts, treatment-related changes in these hematological parameters may influence SII independently of clinical disease activity. Therefore, SII should be interpreted as a marker of systemic inflammatory status rather than a direct surrogate of skin disease severity in all treatment settings. All mean SII and PASI values are reported in Table 3.

### 3.3. Longitudinal Trend of SII

**Figure 2:**
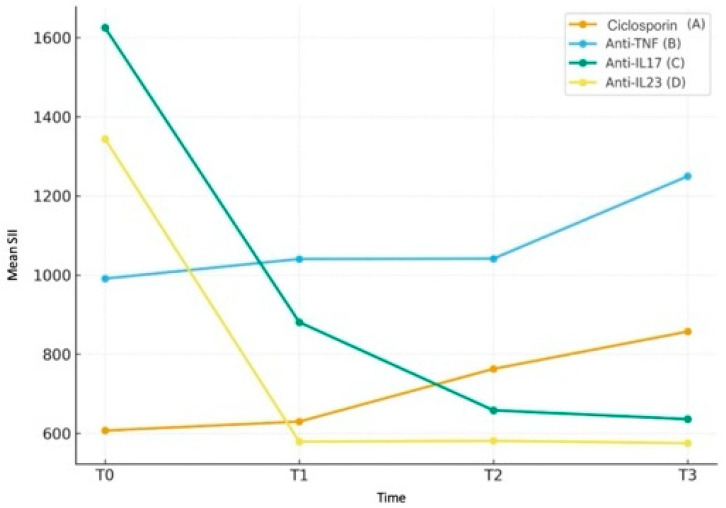
Comparison of the temporal evolution of the Systemic Immune–Inflammation Index (SII) in patients treated with different systemic therapies.

Longitudinal within-group changes in SII were assessed using the Friedman test. Between-group differences in Δ%SII were evaluated using the Kruskal–Wallis test. Visual and descriptive longitudinal trends suggested different SII trajectories across treatment groups. Specifically, mean SII values showed a progressive increase in patients treated with cyclosporine and, to a lesser extent, in those receiving anti-TNF-α therapy. In contrast, patients treated with anti-IL-17 and anti-IL-23 exhibited a marked and sustained reduction in SII over time, highlighting distinct inflammatory response patterns across different therapeutic classes (Figure 2).

**Figure 3:**
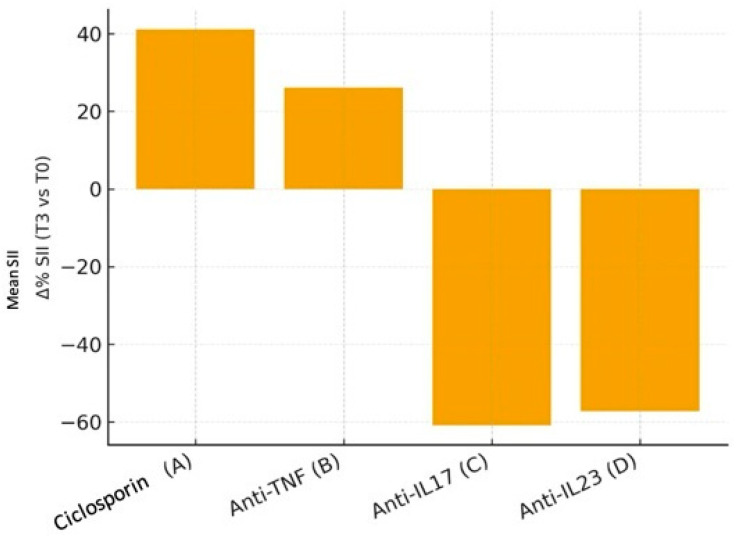
Percentage change in the Systemic Immune–Inflammation Index (SII) in patients treated with different systemic therapies (T3 vs. T0).

Analyses demonstrated statistically significant differences among the four therapeutic groups. Post hoc comparisons using the Dunn–Bonferroni method revealed that patients treated with anti-IL-17 and anti-IL-23 agents experienced a significantly greater and more sustained reduction in SII compared to those receiving cyclosporine or anti-TNF-α therapies (Figure 3). Consistently, mean Δ%SII values indicated an increase in Groups A and B and a marked decrease in Groups C and D. No significant differences were observed within the two clusters (A vs. B and C vs. D), supporting the identification of two distinct anti-inflammatory response profiles: a stable or slightly increasing SII trend in the cyclosporine and anti-TNF-α groups, and a pronounced and persistent reduction in the anti-IL-17 and anti-IL-23 groups (Table 4). To further account for baseline differences among treatment groups, a multivariable linear regression model was performed including age, sex, BMI, psoriatic arthritis, baseline PASI, and treatment group. Age, sex, BMI, psoriatic arthritis, and baseline PASI were not significantly associated with Δ%SII. Compared with cyclosporine, anti-IL-17 (β = −90.7, 95% CI −119.6 to −61.8, *p* < 0.001) and anti-IL-23 therapies (β = −97.9, 95% CI −126.2 to −69.6, *p* < 0.001) were independently associated with greater reductions in SII, whereas anti-TNF therapy showed no significant difference (β = −14.7, 95% CI −37.8 to 8.4, *p* = 0.210) (Table 5).

### 3.4. Subgroup Analysis

To explore whether specific clinical characteristics influenced changes in systemic inflammation, percentage variation in SII (Δ%SII from T0 to T3) was compared across clinically relevant subgroups using the Mann–Whitney U test for dichotomous variables. Subgroups included sex, major comorbidities (psoriatic arthritis, hypertension, and dyslipidemia), and disease localization (nail, palmoplantar, scalp, genital, and pretibial involvement). No statistically significant differences in Δ%SII were observed across any of the comparisons (all *p* > 0.05). These findings indicate that sex, the presence of rheumatologic, cardiovascular, or metabolic comorbidities, and the anatomical distribution of lesions did not significantly influence the evolution of SII during treatment.

## 4. Discussion

This study evaluated the longitudinal behavior of the SII in a cohort of 210 patients with psoriasis undergoing different systemic therapies, with the aim of clarifying its relationship with disease severity and treatment response. At baseline, SII showed a moderate positive correlation with PASI, supporting the link between systemic inflammatory burden and clinical severity, in agreement with previous evidence demonstrating correlations between SII and disease severity as well as other hematological indices [31]. This association weakened during the early phases of treatment and re-emerged at 12 months, suggesting that changes in systemic inflammation may precede and parallel clinical improvement. Such a pattern suggests that SII may represent a useful indicator of systemic inflammatory changes occurring during treatment, capable of capturing hematological normalization before full clinical resolution, consistent with studies showing dynamic variation of blood-derived inflammatory markers during treatment and their association with clinical response [32]. Longitudinal analyses demonstrated that SII trends are strongly influenced by the type of treatment. Patients receiving anti-IL-17 and anti-IL-23 agents exhibited a rapid and sustained reduction in SII, whereas those treated with cyclosporine or anti-TNF-α therapies showed stable or slightly increasing values over time. In contrast, cyclosporine and anti-TNF-α therapies showed stable or increasing SII values over time. This finding may reflect treatment-related effects on the hematological components of SII rather than persistent disease activity. Cyclosporine can influence leukocyte and platelet counts independently of clinical response, whereas TNF inhibition may not uniformly normalize all components included in the SII calculation. These observations support the interpretation of SII as a composite marker of systemic inflammatory status rather than a direct surrogate of skin disease severity. These findings likely reflect differences in mechanisms of action, with interleukin-targeting biologics exerting a more selective and sustained suppression of the IL-23/Th17 axis, a key driver of psoriatic inflammation, in line with studies demonstrating progressive reduction of SII and related indices under IL-17 and IL-23 inhibitors [33]. Consistently, between-group analyses confirmed significant differences in both absolute values and percentage changes of SII, identifying two distinct therapeutic profiles: a limited impact on systemic inflammation for traditional or less targeted therapies, and a marked, durable reduction for selective biologics, as supported by observational data showing significant decreases in SII and other inflammatory indices during biologic therapy [34]. Subgroup analyses did not reveal any significant influence of sex, comorbidities, or disease localization on SII variation, indicating that its trajectory is primarily driven by treatment type and baseline inflammatory status. At the same time, the role of systemic inflammation in psoriasis appears to extend beyond skin involvement, as SII has been associated with metabolic comorbidities and disease burden [13]. In the adjusted multivariable model, treatment class remained the main factor associated with SII variation, whereas age, sex, BMI, psoriatic arthritis, and baseline PASI were not significantly associated with Δ%SII. These findings suggest that differences in SII trajectories were primarily related to the therapeutic class rather than to baseline demographic or clinical characteristics [35]. In contrast, age and baseline PASI did not retain predictive value, highlighting the greater relevance of immuno-hematological parameters over static clinical measures.

Treatment class remained the main factor associated with SII variation, whereas age, sex, BMI, psoriatic arthritis, and baseline PASI were not significant predictors. Importantly, the association between treatment class and SII reduction remained significant after adjustment for age, sex, BMI, psoriatic arthritis, and baseline PASI, suggesting that the observed differences were not solely explained by baseline demographic and clinical imbalances. The main limitations of this study include its retrospective and single-center design, the absence of a healthy control group, and the lack of disease-specific SII cut-off values. An additional limitation is the presence of baseline imbalances among treatment groups, particularly regarding age and prevalence of psoriatic arthritis. These differences reflect treatment selection in routine clinical practice and may have influenced the observed SII trajectories. Therefore, comparisons across treatment classes should be interpreted as descriptive and exploratory rather than causal.

Another limitation is the grouping of different agents within the anti-IL-17 and anti-IL-23 classes. Although these therapies share common immunological targets, they differ in pharmacokinetic properties, dosing schedules, and clinical characteristics. Consequently, pooling individual agents within the same therapeutic class may have masked drug-specific effects on SII trajectories. Additional limitations include the absence of external validation, the inability to establish causal relationships, the lack of information regarding smoking status, alcohol consumption, and previous biologic exposure, and the potential for survivorship bias resulting from the inclusion of patients who maintained the same therapy for 12 months. Nonetheless, the consistency of the findings and the sample size provide a solid basis for future prospective, multicenter studies aimed at validating these results, defining reference thresholds, and comparing SII with other inflammatory markers such as NLR, PLR, C-reactive protein, and IL-6, as well as exploring its prognostic value in long-term outcomes and cardiovascular risk stratification.

## 5. Conclusions

In conclusion, this study suggests that SII may represent a dynamic adjunctive marker of systemic inflammatory changes in psoriasis. Distinct SII trajectories were observed across treatment classes, with greater reductions among patients receiving anti-IL-17 and anti-IL-23 therapies. However, given the retrospective design, baseline imbalances, and absence of validated thresholds, these findings should be interpreted as exploratory. Prospective studies are needed before SII can be recommended for routine clinical decision-making. Overall, SII appears as a dynamic and accessible biomarker reflecting the balance between innate and adaptive immunity, with trends consistent with psoriasis pathophysiology. In this perspective, the use of simple and reproducible blood-based biomarkers like SII represents a step toward a more personalized and comprehensive management of psoriasis, bridging clinical practice and translational research and supporting more informed therapeutic strategies.

## Figures and Tables

**Table 1:** Summary characteristics of the general population. Continuous variables were compared using the Kruskal–Wallis test. Categorical variables were compared using the χ^2^ test. Statistically significant *p*-values are shown in bold.

Variable	General Population (Mean ± SD or %)	Group A N = 45 (Mean ± SD or %)	Group B N = 56 (Mean ± SD or %)	Group C N = 54 (Mean ± SD or %)	Group D N = 55 (Mean ± SD or %)	*p*-Value
Age (years)	49.7 ± 17.5	46 ± 16.7	42 ± 17.2	55 ± 15.3	55 ± 16.4	<0.001
Gender	100 women, 110 men	21 women, 24 men	27 women, 29 men	24 women, 30 men	28 women, 27 men	0.657
BMI	26.4 ± 4.2	25.7 ± 3.7	27.7 ± 4.8	26.4 ± 3.4	25.8 ± 4.2	0.051
Disease duration (years)	16.2 ± 9.5	15.4 ± 10.7	15.6 ± 10	16.4 ± 8.6	17.2 ± 8.7	0.378
Comorbidities:						
PsA	14.3%	0%	23.2%	20.4%	11%	0.004
Hypertension	26.2%	31.1%	26.8%	26%	21.8%	0.772
Diabetes	13.3%	11.1%	8.9%	14.8%	18.2%	0.502
Dyslipidemia	18.1%	11.1%	10.7%	25.9%	23.6%	0.041
Site:						
Nails	37.6%	44.4%	32.1%	35.2%	40%	0.598
Genitals	23.3%	33.3%	17.9%	26.9%	14.55%	0.071
Pretibial	6.7%	13.3%	1.8%	13%	0%	0.005
Palmoplantar	39.5%	35.6%	42.9%	33.3%	45.6%	0.562
Scalp	52.4%	44.4%	57.1%	44.4%	65.6%	0.082

Abbreviations: Standard Deviation (SD); Body Mass Index (BMI); Psoriatic Arthritis (PsA).

**Table 2:** SII–PASI correlations.

Timepoint	Spearman’s Rho	*p*-Value
T0	0.406	<0.001
T1	0.092	0.183
T2	0.108	0.119
T3	0.186	0.007

**Table 3:** Mean SII values and mean PASI values at each timepoint.

	Group A		Group B		Group C		Group D	
	PASI	SII	PASI	SII	PASI	SII	PASI	SII
T0	13.8	607.1	15.5	991	24.2	1625.2	24.1	1344
T1	8.1	629.7	7.8	1040.6	7.9	881	5.6	578.9
T2	3.5	762.9	3.4	1041.5	1.6	658.2	1.1	581.3
T3	1.9	857.2	1.8	1249.7	0.9	635.9	0.4	575.1

Abbreviations: Systemic Immune–Inflammation Index (SII); Psoriasis Area Severity Index (PASI).

**Table 4:** Comparison between treatment groups: percentage change in SII (T3–T0) and results of the Dunn–Bonferroni post hoc test.

Treatment Group	Mean Δ% SII (T3–T0)	SD	Significant Comparison (*p* < 0.05)
A—Cyclosporine	+41.2%	±18.7%	vs. C (*p* < 0.001); vs. D (*p* < 0.001)
B—Anti-TNF-α	+25.9%	±22.3%	vs. C (*p* < 0.001); vs. D (*p* < 0.001)
C—Anti-IL-17	−60.9%	±24.6%	vs. A (*p* < 0.001); vs. B (*p* < 0.001)
D—Anti-IL-23	−66.1%	±21.8%	vs. A (*p* < 0.001); vs. B (*p* < 0.001)

**Table 5:** Multivariable linear regression analysis of factors associated with Δ%SII.

Variable	β Coefficient	95% CI	*p*-Value
Age	−0.25	−0.71 to 0.21	0.279
Male sex	−9.25	−24.82 to 6.33	0.243
BMI	1.08	−0.81 to 2.97	0.263
PsA	−4.82	−27.26 to 17.63	0.673
Baseline PASI	−0.68	−2.36 to 1.01	0.429
Anti-TNF vs. cyclosporine	−14.71	−37.80 to 8.38	0.210
Anti-IL-17 vs. cyclosporine	−90.73	−119.64 to −61.82	<0.001
Anti-IL-23 vs. cyclosporine	−97.90	−126.16 to −69.64	<0.001

## Data Availability

The data that support the findings of this study are available from the corresponding author upon reasonable request.
